# Effectiveness of Pelvic Floor Muscle Training in Preventing Urinary Incontinence After Vaginal Delivery: A Systematic Review

**DOI:** 10.7759/cureus.88059

**Published:** 2025-07-16

**Authors:** Eman Mohammed Abbashar Abdelmahmoud, Nashwa Fathelrahman Elawad Ahmed, Samah Mohamed Yousif Idris, Raim Chaar, Ahmed Mohamed Ali Abdelsalam, Rania Elrasheed Belal Mohammed

**Affiliations:** 1 Obstetrics and Gynecology, Sultan Qaboos Hospital, Salalah, OMN; 2 Obstetrics and Gynecology, Alzaiem Alazhari University, Khartoum, SDN; 3 Obstetrics and Gynecology, King Faisal Specialist Hospital and Research Centre, Riyadh, SAU; 4 Obstetrics and Gynecology, Nottingham University Hospitals NHS Trust - Queen's Medical Centre, Nottingham, GBR; 5 Obstetrics and Gynecology, Cavan General Hospital, Cavan, IRL; 6 Obstetrics and Gynecology, Mouwasat Hospital, Jubail, SAU

**Keywords:** pelvic floor muscle training, postpartum, prevention, systematic review, urinary incontinence, vaginal delivery

## Abstract

Urinary incontinence (UI) is a common issue among women after vaginal delivery and can have various impacts on daily life. Pelvic floor muscle training (PFMT) is often used as a preventive intervention, although its effectiveness has shown mixed results in research. This systematic review evaluates the effectiveness of PFMT in preventing UI after vaginal delivery by synthesizing evidence from randomized controlled trials (RCTs). Following the Preferred Reporting Items for Systematic reviews and Meta-Analyses (PRISMA) 2020 guidelines, multiple databases were searched for relevant RCTs published before May 2025. Eight studies met the inclusion criteria and were assessed for risk of bias using the Cochrane risk of bias assessment tool 2 (RoB 2). Due to methodological differences, data were narratively synthesized. Overall, PFMT showed benefits in reducing UI and improving quality of life, particularly when delivered with structured support. However, some studies reported no significant effects. Adherence emerged as an important factor influencing outcomes. Most studies had a low risk of bias, though feasibility trials showed higher risks. PFMT appears to be a promising approach for preventing postpartum UI, especially with supervised or technology-assisted protocols. Further research is needed to standardize interventions, ensure long-term follow-up, and evaluate implementation across various healthcare settings.

## Introduction and background

Urinary incontinence (UI) is a highly prevalent condition among postpartum women, particularly following vaginal delivery, with substantial implications for physical health, psychological well-being, and overall quality of life [1]. Defined as the involuntary loss of urine, UI is commonly classified into stress, urge, and mixed incontinence, with stress UI (SUI) being the most frequent subtype in the postpartum population [2]. Epidemiological studies indicate that approximately 30%-50% of women may experience some form of urinary leakage after childbirth, with vaginal delivery recognized as a significant risk factor due to its mechanical and neurological impact on the pelvic floor musculature [3].

The pelvic floor is a complex anatomical and functional structure comprising muscles, fascia, and connective tissue, which together provide support to the pelvic organs and contribute to urinary continence [4]. During vaginal delivery, the integrity of the pelvic floor is frequently compromised through muscle overstretching, nerve injury, and tissue trauma [5]. These physiological alterations can lead to weakened pelvic floor muscle (PFM) tone and coordination, thereby increasing susceptibility to urinary leakage [6]. Given the multifactorial etiology of postpartum UI, there is increasing emphasis on early, non-invasive interventions that can mitigate or prevent the onset of symptoms [7].

PFM training (PFMT), commonly known as Kegel exercises, is a conservative, low-risk intervention designed to enhance the strength, endurance, and neuromuscular control of the PFMs [8]. Originally popularized in the mid-20th century, PFMT has evolved into a cornerstone of preventive and therapeutic strategies for UI [9], supported by growing evidence from randomized controlled trials (RCTs) and clinical guidelines. The underlying principle of PFMT is to restore the structural and functional capacity of the pelvic floor, thereby improving urethral closure pressure and bladder support during increased intra-abdominal pressure events, such as coughing or physical exertion [10].

Despite widespread clinical endorsement of PFMT, the strength and consistency of evidence regarding its preventive effectiveness for UI specifically after vaginal delivery remain variable. Disparities in study design, training protocols, timing and intensity of intervention, and outcome measurement tools have contributed to inconsistent findings across the literature. Furthermore, adherence to exercise regimens and variations in the implementation of supervised versus unsupervised training introduce additional methodological complexity. As a result, there remains a critical need to synthesize the available evidence through rigorous systematic review methods to inform clinical decision-making and optimize postpartum rehabilitation protocols.

This systematic review aims to critically evaluate the effectiveness of PFMT in preventing UI among women following vaginal delivery. By consolidating data from high-quality studies conducted over the past five years, this review seeks to elucidate the preventive value of PFMT, identify gaps in current research, and offer recommendations for future clinical practice and policy development.

## Review

Methodology

Eligibility Criteria

This systematic review was conducted in accordance with the Preferred Reporting Items for Systematic Reviews and Meta-Analyses (PRISMA) 2020 guidelines [11]. Studies were included if they met the following criteria: (1) RCTs, quasi-experimental studies, or controlled clinical trials investigating the effectiveness of PFMT in preventing UI after vaginal delivery; (2) participants were postpartum women following vaginal birth without prior history of urinary incontinence; (3) the intervention involved PFMT as a preventive strategy, either supervised or unsupervised; (4) outcomes measured included incidence or prevalence of postpartum UI, or validated continence assessment scores; and (5) articles published before May 2025. Studies focusing on treatment rather than prevention, those involving cesarean sections exclusively, or those lacking a comparator group were excluded.

Information Sources and Search Strategy

An extensive and systematic search of the literature was conducted across five electronic databases: PubMed/MEDLINE, Scopus, Web of Science, Cochrane Library, and CINAHL. The search covered articles published before May 2025. The search strategy combined Medical Subject Headings (MeSH) and free-text terms related to pelvic floor muscle training, urinary incontinence, postpartum or postnatal period, and vaginal delivery. Reference lists of included articles and relevant reviews were manually screened for additional eligible studies. The search strategy for PubMed is shown in Table 1.

**Table 1:** PubMed search strategy

Search String	Description
("pelvic floor muscle training"[MeSH Terms] OR "pelvic floor exercises" OR "Kegel exercises" OR "PFMT") AND ("urinary incontinence"[MeSH Terms] OR "postpartum urinary incontinence" OR "UI") AND ("postpartum period"[MeSH Terms] OR "postnatal" OR "vaginal delivery" OR "childbirth") AND ("prevention and control"[Subheading] OR "preventive")	Search string combining intervention, outcome, population, and purpose

Search strategies were adapted for each database using controlled vocabulary (e.g., MeSH or CINAHL headings) and keyword combinations appropriate for that platform. All search results were exported into a citation management tool, and duplicates were removed using automated and manual checks.

Study Selection

All identified records were screened in two stages. First, titles and abstracts were independently reviewed by two reviewers (AMAA and RC) from the list of authors to identify potentially relevant studies. Disagreements were resolved through discussion or the involvement of a third reviewer (SMYR). In the second stage, full-text articles of shortlisted studies were assessed in detail based on the eligibility criteria. Studies that did not meet the inclusion criteria at this stage were documented with reasons for exclusion. The final selection was documented using the Preferred Reporting Items for Systematic reviews and Meta-Analyses (PRISMA) 2020 flow diagram.

Data Extraction

Data from the included studies were extracted independently by two reviewers (AMAA and RC) using a standardized data extraction form. Extracted variables included author(s), year of publication, country, study design, sample size, participant characteristics, timing of PFMT initiation, type and frequency of intervention, comparator details, duration of follow-up, outcome measures, and key findings related to UI prevention. Discrepancies in extracted data were resolved through consensus or third-person (SMYR) adjudication.

Risk of Bias Assessment

The risk of bias in the included RCTs was assessed using the Revised Cochrane risk-of-bias tool for randomized trials (RoB 2) [12]. This tool evaluates five domains: bias arising from the randomization process, deviations from intended interventions, missing outcome data, measurement of the outcome, and selection of the reported result. Each domain was rated as "low risk," "some concerns," or "high risk." Two independent reviewers performed the assessments, and any disagreements were reconciled through discussion.

Data Synthesis

Given the heterogeneity in the PFMT protocols, timing of intervention initiation, outcome measurement tools, and duration of follow-up across studies, it was determined that a meta-analysis was not methodologically appropriate. Specifically, variations in the intensity and supervision of PFMT programs, inconsistent use of validated outcome measures (e.g., International Consultation on Incontinence Questionnaire - Urinary Incontinence Short Form (ICIQ-UI-SF), pad tests, self-reported symptoms), and differences in defining urinary incontinence across studies precluded the possibility of pooling data reliably. Therefore, a narrative synthesis approach was employed to summarize and interpret the findings. Patterns and trends in intervention effectiveness were reported descriptively, and emphasis was placed on the methodological quality and contextual relevance of the studies.

Results

Search Results

**Figure 1:**
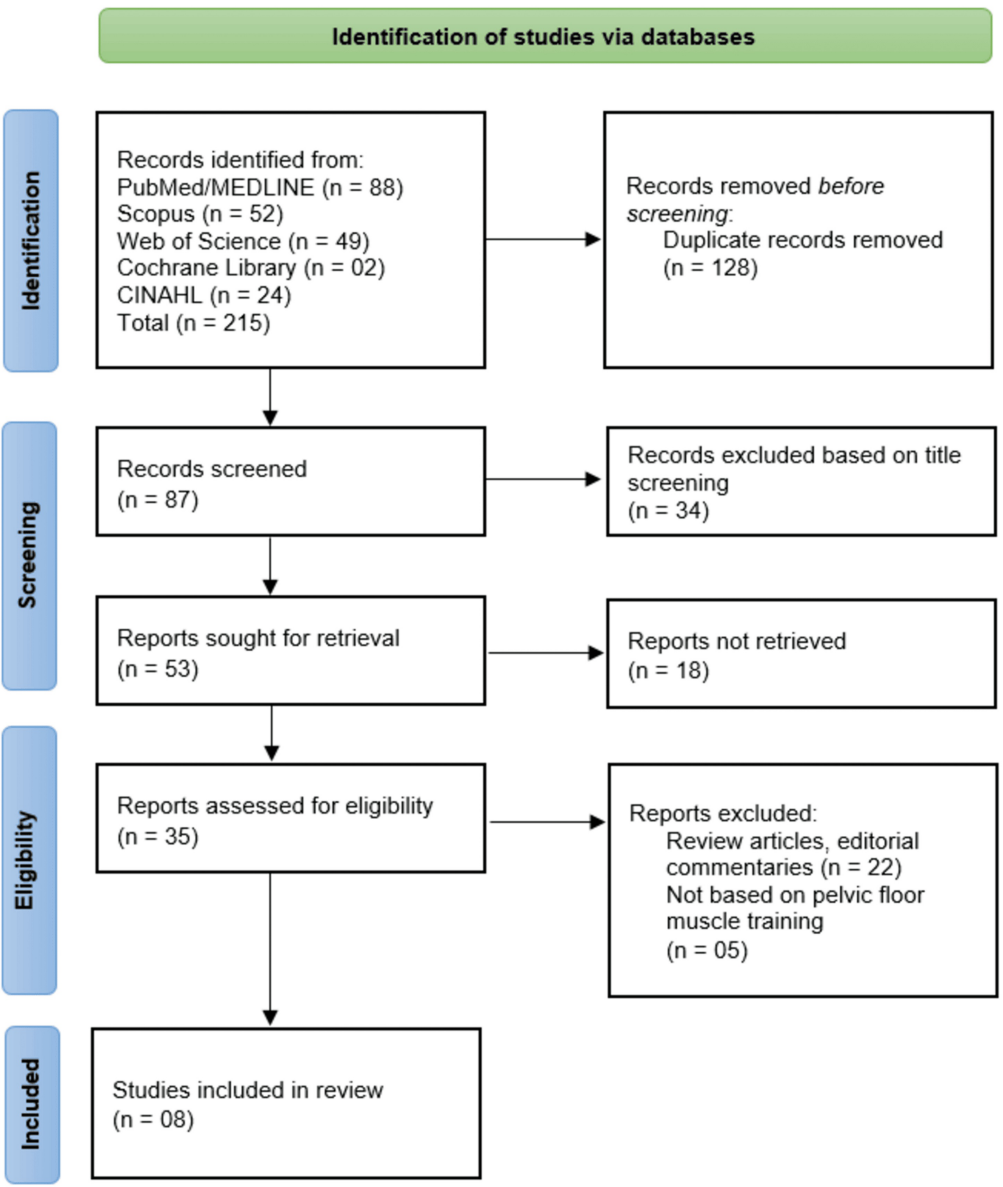
Study selection flowchart based on the PRISMA framework for this systematic review PRISMA: Preferred Reporting Items for Systematic reviews and Meta-Analyses

The systematic search across five databases (PubMed/MEDLINE, Scopus, Web of Science, Cochrane Library, and CINAHL) initially identified 215 records, from which 128 duplicates were removed before screening. The remaining 87 records underwent title screening, resulting in the exclusion of 34 irrelevant studies. A total of 53 full-text articles were sought for retrieval, but 18 were unavailable, leaving 35 reports for eligibility assessment. Among these, 22 were excluded as review articles or editorial commentaries, and five were excluded for not focusing on PFMT. Ultimately, eight studies [13-20] met the inclusion criteria and were incorporated into the systematic review (Figure 1).

Characteristics of the Included Studies

The systematic review included eight RCTs [13-20] that evaluated the effectiveness of PFMT in preventing UI after vaginal delivery. The studies were conducted across diverse geographical regions, including Thailand, China, the United Kingdom, France, Poland, and Norway. Sample sizes ranged from 48 to 452 participants, with populations primarily consisting of nulliparous or primiparous pregnant or postpartum women. The timing of PFMT interventions varied, with some studies initiating training during pregnancy [13,18] and others postpartum [14,20]. Intervention designs included supervised PFMT, home-based training, smartphone reminders, biofeedback devices, and group-based sessions. Comparators typically involved usual antenatal care, written instructions, or standard PFMT without additional support (Table 2).

**Table 2:** Characteristics of the included studies EMG: electromyography; ICIQ-UI SF: International Consultation on Incontinence Questionnaire - Urinary Incontinence Short Form; LUTS: lower urinary tract symptoms; PGI-I: patient global impression of improvement; PFMT: pelvic floor muscle training; QoL: quality of life; RCT: randomized controlled trial; UI: urinary incontinence

Author (Year)	Country	Study Design	Sample Size	Population Characteristics	Timing of Intervention	Type of Intervention (PFMT)	Comparator	Duration of Follow-Up	Outcome Measures	Key Findings
Jinapun et al., [13] (2024)	Thailand	RCT	131 (PFMT: 63; control: 68)	Singleton nulliparous women without pre-existing UI	Late third trimester (36–38 weeks gestation)	Video education + PFMT instruction (muscle contraction training)	Usual antenatal care (standard routine care)	Until late third trimester before delivery	UDI-6 score, IIQ-7 score, prevalence of UI	PFMT group showed significantly lower UI prevalence (20.6% vs 94.1%), lower UDI-6 (5.5 vs 27.7), and lower IIQ-7 (0 vs 14.3); PFMT improved UI prevention and QoL
Chu et al., [14] (2024)	China	RCT	148 (76 intervention, 72 control)	Primiparous women, 6 weeks postpartum, admitted to Tongji Hospital	6 weeks postpartum	PFMT with daily smartphone reminders (via WeChat)	PFMT without smartphone reminders	3 months	PFMT adherence, stress UI symptoms, PFM surface EMG, PFM endurance, ICIQ-SF, PGI-I	Higher PFMT adherence and improved PFM EMG and endurance in the intervention group; no significant difference in SUI symptoms (ICIQ-SF or PGI-I scores) between groups.
Wang et al., [15] (2024)	China	Multicenter assessor-blinded RCT	452 (223 intervention, 229 control)	Women with new-onset postpartum SUI; median age: 34 years; median BMI: 23.71; median: 50 days postpartum	Median: 50 days postpartum	Supervised PFMT + home-based pressure-mediated biofeedback device	Home-based PFMT alone	3 months	ICIQ-UI SF score, cure rate, improvement rate, PFM strength, QoL, self-efficacy, adherence	Intervention group showed significantly greater improvement in all primary and secondary outcomes compared to control (e.g., higher cure rate, greater PFM strength, and better adherence)
Yang et al., [16] (2025)	China	Feasibility RCT (accompanied by mixed methods process evaluation)	48	Pregnant women	During pregnancy	Group-based supervised PFMT, developed using MRC framework and behaviour change wheel, delivered in group setting	NR	NR	Feasibility (recruitment and adherence rate), acceptability of the program	Recruitment rate: 52.17%, adherence rate: 66.67%; intervention perceived as acceptable and supportive; indicated potential for reducing UI and feasible delivery using limited health resources
Mason et al., [17] (2010)	United Kingdom	RCT	286 (141 intervention, 145 control)	Primiparous pregnant women recruited from two hospitals in north-west England	Antenatal (20–36 weeks of gestation)	4 sessions of taught PFMT during pregnancy + home-based PFMT (8–12 maximal contractions twice daily)	Usual care (no formal PFMT sessions)	3 months postpartum	Modified Bristol Female LUTS Questionnaire, Leicester Impact Scale, Three Day Diary	No statistically significant differences in incontinence or bother; however, intervention group reported fewer episodes and lower bother scores.
Fritel et al., [18] (2015)	France	RCT	282	Nulliparous pregnant women recruited during 2nd trimester	During pregnancy (prenatal)	Supervised individual PFMT + written instructions	Written instructions only (home exercises)	12 months postpartum	Primary: UI severity (ICIQ-UI SF score); Secondary: UI prevalence, pelvic floor troubles (self-administered questionnaires)	No statistically significant difference in UI severity, prevalence, or pelvic floor troubles at any time point
Dornowski et al., [19] (2018)	Poland	Quasi-experimental RCT	113	Pregnant women with and without symptoms of stress UI (SUI)	During pregnancy (not explicitly stated)	6-week pelvic floor muscle training program	Control group (no SUI symptoms or no intervention—unclear)	6 weeks	PFM EMG activity (BASE and R values), Incontinence Impact Questionnaire (IIQ)	PFMT led to increased BASE values post-training in symptomatic and control groups. R values decreased after training. Indicates improved PFM activity post-intervention but reduced control during prolonged contractions in symptomatic participants.
Hilde et al., [20] (2013)	Norway	Assessor-blinded RCT	175 (87 intervention, 88 control)	Primiparous women, 6 weeks postpartum; stratified by presence/absence of major levator ani muscle defects	6 weeks postpartum	Weekly supervised PFMT + daily home exercises for 16 weeks	Standard care (all taught to contract pelvic floor muscles; control received no further intervention)	6 months postpartum	Self-reported UI	No statistically significant difference in UI prevalence between groups (RR = 0.89, 95% CI 0.60–1.32); similar nonsignificant results in both stratified subgroups

Effectiveness of PFMT in Preventing UI

The outcomes of PFMT interventions demonstrated mixed results across the included studies. Jinapun et al. [13] reported significant reductions in UI prevalence (20.6% vs. 94.1%), lower Urogenital Distress Inventory (UDI-6) scores (5.5 vs. 27.7), and improved quality of life (Incontinence Impact Questionnaire (IIQ-7) scores: 0 vs. 14.3) among nulliparous women who received PFMT during the third trimester compared to usual care (p < 0.001). Similarly, Wang et al. [15] found that supervised PFMT combined with pressure-mediated biofeedback significantly improved cure rates (20.2% vs. 8.7%), PFM strength (median 26.00 vs. 21.00 cm H₂O), and adherence (r = 0.825 vs. 0.627) compared to home-based PFMT alone (p ≤ 0.02 for all outcomes).

However, other studies reported no statistically significant differences in UI outcomes. Fritel et al. [18] observed no reduction in UI severity (mean ICIQ-UI SF score: 1.9 vs. 2.1) or prevalence at 12 months postpartum (p = 0.38) despite supervised prenatal PFMT. Hilde et al. [20] also found no significant difference in UI prevalence between the intervention and control groups (relative risk (RR) = 0.89, 95% CI: 0.60-1.32), even when stratified by levator ani muscle defects. Mason et al. [17] noted fewer self-reported incontinence episodes and lower bother scores in the PFMT group, but these differences were not statistically significant in objective measures (p > 0.05).

Adherence and Feasibility of PFMT Interventions

Adherence to PFMT and feasibility of delivery were highlighted as critical factors influencing outcomes. Chu et al. [14] demonstrated that smartphone reminders significantly improved adherence rates (53.9% vs. 20.8%; p = 0.00) and PFM electromyography (EMG) activity (p = 0.03) compared to standard PFMT, though no improvements were observed in stress UI symptoms (ICIQ-SF: p = 0.60). Yang et al. [16] reported a 66.67% adherence rate and high acceptability for group-based PFMT among pregnant women, suggesting its feasibility in low-resource settings. Conversely, Dornowski et al. [19] found that a six-week PFMT program improved PFM EMG activity (BASE values) but noted reduced control during sustained contractions (R values), indicating variability in long-term efficacy.

Summary of Key Findings

The results of this systematic review suggest that PFMT can be effective in reducing UI prevalence and improving quality of life, particularly when delivered with structured support (e.g., biofeedback, group sessions, or reminders). However, its effectiveness may depend on intervention timing, adherence, and population characteristics, as some studies reported null findings. Table 3 summarizes the heterogeneity in outcomes, emphasizing the need for standardized protocols and long-term follow-up to clarify PFMT's role in UI prevention post-vaginal delivery.

**Table 3:** Summary of intervention outcomes PFM: Pelvic floor muscle

Author (Year)	Intervention Details	Comparator	Outcome Measure(s)	Timing of Outcome Assessment	Effectiveness Outcome	Statistical Significance (p-value/CI)
Jinapun et al., [13] (2024)	Video education and instruction on pelvic floor muscle contraction during antenatal care (PFMT group, n=63)	Usual antenatal care (n=68)	Urogenital Distress Inventory (UDI-6); Incontinence Impact Questionnaire (IIQ-7); prevalence of UI	36–38 weeks of gestation (late third trimester)	Lower prevalence of UI (20.6% vs. 94.1%); lower UDI-6 score (5.5 vs. 27.7); lower IIQ-7 score (0 vs. 14.3)	All outcomes: p < 0.001
Chu et al., [14] (2024)	Pelvic Floor Muscle Training (PFMT) with daily smartphone reminders via WeChat	Standard PFMT without smartphone reminders	Adherence to PFMT; peak surface EMG of PFMs; PFM endurance; ICIQ-SF scores; PGI-I scores	3 months postpartum	Higher adherence (53.9% vs. 20.8%); improved EMG (39.8 ± 6.2 vs. 37.5 ± 5.9 μV); longer PFM endurance (8.1 ± 2.0 vs. 7.3 ± 2.0 sec); no improvement in ICIQ-SF or PGI-I	Adherence: p = 0.00; EMG: p = 0.03- Endurance: p = 0.01; ICIQ-SF: p = 0.60; PGI-I: p = 1.00
Wang et al., [15] (2024)	Supervised Pelvic Floor Muscle Training (PFMT) + Home-based Pressure-Mediated Biofeedback (BF) Device	Home-based PFMT alone	International Consultation on Incontinence Questionnaire; Urinary Incontinence Short Form (ICIQ-UI SF) score; Cure and improvement rates; Pelvic Floor Muscle (PFM) strength (cm H₂O); quality of life, self-efficacy, adherence (subjective and objective)	After 3 months of supervised PFMT	Greater reduction in incontinence severity (median: 3.00 vs. 2.00); higher cure rate (20.2% vs. 8.7%); higher improvement rate (59.2% vs. 44.5%); greater PFM strength (median: 26.00 vs 21.00 cm H₂O); higher adherence correlation (r = 0.825 vs. 0.627)	ICIQ-UI SF: p = 0.002; Cure rate: p = 0.001; improvement rate: p = 0.002; PFM strength: p = 0.02
Yang et al., [16] (2025)	Group-based pelvic floor muscle training for pregnant women in China.	NR	Feasibility (recruitment and adherence rates), acceptability (qualitative feedback), and potential to reduce UI prevalence	Post-intervention (after completion of training)	66.67% adherence rate; positive acceptability reported; suggested potential to reduce urinary incontinence in pregnant women	NR
Mason et al., [17] (2010)	Four taught sessions of pelvic floor muscle training during pregnancy + 8–12 maximal contractions twice daily at home	Routine antenatal care (no structured PFMT training)	Modified Bristol Female Lower Urinary Tract Symptom questionnaire, Leicester Impact Scale, Three-Day Diary	20 weeks, 36 weeks of pregnancy, and 3 months postpartum	Intervention group reported fewer episodes of incontinence and lower symptom impact, but no significant difference in actual incontinence episodes or bother postpartum	PFMT adherence higher at 36 weeks (p = 0.019) and 3 months (p = 0.022); differences in primary outcomes not statistically significant
Fritel et al., [18] (2015)	Prenatal individually supervised pelvic floor muscle training + written instructions	Written instructions only	UI severity (ICIQ-UI SF score); UI prevalence; pelvic floor troubles (self-administered questionnaire)	Baseline, end of pregnancy, 2 months postpartum, 12 months postpartum	No significant difference in UI severity (mean score: 1.9 vs. 2.1), UI prevalence, or pelvic floor troubles at any time point	p = 0.38 for primary outcome (UI severity at 12 months)
Dornowski et al., [19] (2018)	6-week pelvic floor muscle training (PFMT); assessed using PFM EMG and Incontinence Impact Questionnaire (IIQ)	Control group (no PFMT) and baseline (pre-intervention) measurements	PFM EMG activity (BASE and R values), IIQ score	At baseline and after 6 weeks of PFMT	Increased BASE values post-training in all groups; R values decreased or remained unchanged, indicating reduced control during sustained contractions	Statistically significant changes observed (exact p-values not provided, described as "statistically significant differences")
Hilde et al., [20] (2013)	Weekly supervised pelvic floor muscle training + daily home exercises for 16 weeks, beginning 6 weeks postpartum. All participants taught to contract PFM	No further intervention beyond initial instruction to contract PFM	Self-reported urinary incontinence (UI)	6 months after delivery	34.5% in the training group vs. 38.6% in the control group reported UI. Relative risk (RR) = 0.89 (training vs. control), indicating a non-significant difference	RR = 0.89, 95% CI: 0.60–1.32 (overall); with defects: 0.89, CI: 0.51–1.56; without defects: 0.90, CI: 0.53–1.52

Risk of Bias Results

The risk of bias assessment, conducted using the Cochrane RoB 2 tool, revealed that most studies had a low overall risk of bias, including Jinapun et al. [13], Chu et al. [14], Wang et al. [15], Mason et al. [17], Fritel et al. [18], and Hilde et al. [20], as they demonstrated low risk across all domains, such as randomization, deviations from interventions, missing data, outcome measurement, and reporting. However, Yang et al.'s [16] study was rated as high risk due to some concerns in randomization and missing data, along with high risk in outcome measurement, given its feasibility design and qualitative focus. Similarly, Dornowski et al.'s [19] study was deemed high risk due to some concerns in randomization and missing data, coupled with high risk in deviations from intended interventions and some concerns in outcome measurement and reporting, reflecting its quasi-experimental limitations (Table 4). Overall, the majority of studies were methodologically robust, though a few exhibited notable biases.

**Table 4:** Risk of bias assessment using Cochrane RoB 2 tool

Study (Author, Year)	Randomization Process	Deviations from Intended Interventions	Missing Outcome Data	Measurement of the Outcome	Selection of Reported Result	Overall Risk of Bias
Jinapun et al., [13] (2024)	Low risk	Low risk	Low risk	Low risk	Low risk	Low risk
Chu et al., [14] (2024)	Low risk	Low risk	Low risk	Low risk	Low risk	Low risk
Wang et al., [15] (2024)	Low risk	Low risk	Low risk	Low risk	Low risk	Low risk
Yang et al., [16] (2025)	Some concerns	Some concerns	Some concerns	High risk	Some concerns	High risk
Mason et al., [17] (2010)	Low risk	Low risk	Low risk	Low risk	Low risk	Low risk
Fritel et al., [18] (2015)	Low risk	Low risk	Low risk	Low risk	Low risk	Low risk
Dornowski et al., [19] (2018)	Some concerns	High risk	Some concerns	Some concerns	Some concerns	High risk
Hilde et al., [20] (2013)	Low risk	Low risk	Low risk	Low risk	Low risk	Low risk

Discussion

The findings of this systematic review provide a comprehensive synthesis of the current evidence on the effectiveness of PFMT in preventing UI after vaginal delivery. The review included eight RCTs conducted across diverse populations and settings, revealing both promising outcomes and notable inconsistencies. The pooled evidence suggests that PFMT, particularly when delivered with structured support such as biofeedback, group sessions, or digital reminders, can significantly reduce UI prevalence and improve quality of life in postpartum women. For instance, Jinapun et al. [13] demonstrated a dramatic reduction in UI prevalence (20.6% vs. 94.1%) and lower distress scores (UDI-6: 5.5 vs. 27.7) among nulliparous women who received antenatal PFMT compared to usual care. Similarly, Wang et al. [15] reported superior outcomes with supervised PFMT combined with biofeedback, including higher cure rates (20.2% vs. 8.7%) and improved PFM strength. These findings align with existing meta-analyses, such as Boyle et al. [21], which concluded that supervised PFMT is more effective than unsupervised training in reducing postpartum UI. However, the heterogeneity in intervention designs and outcomes across studies underscores the need for cautious interpretation.

Despite these positive results, several studies in this review reported null findings, highlighting the complexity of PFMT’s effectiveness. For example, Fritel et al. [18] found no significant differences in UI severity or prevalence at 12 months postpartum despite supervised prenatal PFMT, while Hilde et al. [20] observed no reduction in UI prevalence (RR = 0.89, 95% CI: 0.60-1.32) even with postpartum training. These discrepancies may stem from variations in intervention timing, adherence rates, or population characteristics. Mason et al. [17] noted that, while PFMT reduced self-reported bother, it did not significantly alter objective incontinence measures, suggesting that perceived benefits may not always correlate with clinical outcomes. Such mixed results mirror those of Dumoulin et al. [22], whose Cochrane review emphasized that PFMT’s efficacy depends heavily on proper technique adherence and individualized supervision. The inclusion of feasibility studies, such as Yang et al. [16], which reported high acceptability but limited generalizability, further complicates the evidence landscape.

Adherence emerged as a critical moderator of PFMT success, a theme consistent with broader literature. Chu et al. [14] demonstrated that smartphone reminders doubled adherence rates (53.9% vs. 20.8%) and improved PFM electromyography (EMG) activity, though without impacting UI symptoms. This aligns with innovations in digital health, where tools such as mobile apps have shown promise in enhancing compliance [23]. Conversely, Dornowski et al. [19] found that even a six-week PFMT program improved EMG activity but failed to sustain muscle control during prolonged contractions, suggesting that short-term interventions may lack durability. These insights resonate with Woodley et al. [24], who argued that long-term PFMT regimens are essential for sustained benefits. The feasibility of group-based PFMT, as explored by Yang et al. [16], offers a pragmatic solution for resource-limited settings, though its scalability requires further investigation.

The risk of bias assessment revealed methodological strengths in most studies, with six out of eight rated as low risk. However, exceptions such as Yang et al. [16] and Dornowski et al. [19], which had high risk due to feasibility designs and quasi-experimental limitations, caution against overgeneralizing their findings. The predominance of self-reported outcomes in studies such as Hilde et al. [20] and Fritel et al. [18] also introduces potential measurement bias, a common critique in UI research [25]. Nonetheless, the robust methodologies of studies such as Wang et al. [15] - assessor-blinded with objective measures - strengthen confidence in their conclusions.

When contextualized within existing literature, this review’s findings both corroborate and challenge prior syntheses. The superiority of supervised, biofeedback-enhanced PFMT aligns with Burton et al. [26], who reported greater efficacy with biofeedback in strengthening PFM. However, the lack of significant effects in some studies contrasts with Mørkved et al. [27], who found consistent UI reduction with antenatal PFMT. This discrepancy may reflect differences in intervention intensity or follow-up duration. Notably, the review’s emphasis on adherence mirrors the WHO’s [28] guidelines on exercise interventions, which stress behavioral support for long-term habit formation.

Limitations

This review has several limitations. First, the heterogeneity in intervention designs, populations, and outcome measures precluded meta-analysis, limiting quantitative synthesis. Second, the predominance of studies from high-income settings (e.g., China, Norway) may restrict generalizability to low-resource contexts. Third, the reliance on self-reported outcomes in some studies introduces potential bias. Finally, the lack of long-term follow-up in most trials (e.g., 3-12 months) leaves the durability of PFMT effects uncertain.

## Conclusions

The battle against postpartum urinary incontinence may have found its most powerful weapon yet - pelvic floor muscle training. This systematic review reveals that structured PFMT programs, especially when enhanced with supervision or technology, can be a game-changer for new mothers, dramatically reducing incontinence rates and boosting quality of life. However, the solution is not one-size-fits-all; while some women experience life-changing improvements, others see modest benefits, highlighting the crucial role of personalized approaches and consistent adherence. What is the key takeaway? PFMT works, but its success hinges on how it is delivered. As we move forward, the focus must shift to refining these interventions - standardizing protocols, extending follow-up periods, and making them accessible across diverse healthcare settings. The potential is undeniable: with the right strategy, PFMT could transform postpartum recovery for millions of women worldwide. The time to act on this evidence is now.
